# The role of co-occurring obesity in the association between lumbar disc degeneration and disability related to low back pain

**DOI:** 10.1186/s12891-026-09514-5

**Published:** 2026-01-19

**Authors:** Teija Mertimo, Petteri Oura, Jaro Karppinen, Jaakko Niinimäki, Roberto Blanco Sequeiros, Juhani Määttä, Markku Kankaanpää, Eveliina Heikkala

**Affiliations:** 1 Faculty of Medicine and Health Technology, Tampere University Hospital and University of Tampere, P.O. Box 607, Tampere, FI-33014 Finland; 2 Research Unit of Health Sciences and Technology, Faculty of Medicine, University of Oulu, P.O. Box 5000, Oulu, FI-90014 Finland; 3 Medical Research Center Oulu, Oulu University Hospital and University of Oulu, P.O. Box 5000, Oulu, FI-90014 Finland; 4 Rehabilitation Services of South Karelia Social and Health Care District, Valto Käkelän katu 3, Lappeenranta, FI-53130 Finland; 5 Department of Radiology, Turku University Hospital and Turku University, Kiinamyllynkatu 4-8, FI-20520 Turku, Finland; 6 Department of Rehabilitation and Psychosocial Support, Tampere University Hospital, P.O. Box 2000, Tampere, FI-33521 Finland; 7 Wellbeing Service County of Lapland, Rovaniemi, Finland

**Keywords:** Obesity, Lumbar disc degeneration, Low back pain, Magnetic resonance imaging, Pain-related disability, Prevalence, Finland, Northern Finland Birth Cohort 1966

## Abstract

**Background:**

Low back pain (LBP) and obesity-related diseases cause a significant burden to both individuals and societies. Although not confirmed by all studies, a significant association has been found between lumbar disc degeneration (LDD) and LBP. The role of obesity in this association is not known. Our aim was to investigate whether obesity, measured by different indicators, modifies the association between LDD and LBP-related disability.

**Methods:**

A total of 1080 individuals who had experienced LBP during the previous year responded to questionnaires, participated in a clinical examination, and underwent 1.5-T lumbar magnetic resonance imaging at the age of 47. Full data were available for 842 individuals. LBP-related disability (numerical rating scale, range 0–10) was assessed as the outcome. LDD was evaluated by a Pfirrmann-based sum score (range 0–15) and was assessed as the exposure. As regards outcome and exposure, higher values reflected a greater disability or LDD burden, respectively. The role of obesity (according to five different anthropometric indicators) in the association between the LDD sum score and LBP-related disability was analysed using general linear regression models stratified by the presence of obesity. Adjustments were made for sex, smoking, education, leisure-time physical activity, occupational physical exposure, Modic changes and disc herniations.

**Results:**

A significant positive association between LDD and LBP-related disability was observed among the individuals without obesity, regardless of the anthropometric indicator used: body mass index (adjusted beta [ß] = 0.119, 95% confidence interval [Cl] = 0.030–0.208, *p* = 0.009), waist circumference (0.134, 0.038–0.231, *p* = 0.007), body fat percentage (0.187, 0.091–0.283, *p* < 0.001), waist-to-height ratio (0.149, 0.064–0.235, *p* < 0.001), and waist-to-hip ratio (0.171, 0.067–0.275, *p* = 0.001). No significant associations were detected between LDD and LBP-related disability among the individuals with obesity.

**Conclusions:**

LDD is associated with LBP-related disability among individuals without obesity but not among those with obesity. Although this cross-sectional study cannot establish causality, the findings suggest that LBP-related disability may be related also to factors other than LDD burden among individuals with obesity. This study adds to the evidence that obesity may modify the association between LDD and LBP-related disability.

**Supplementary Information:**

The online version contains supplementary material available at 10.1186/s12891-026-09514-5.

## Background

Low back pain (LBP) and obesity-related diseases such as circulatory diseases and osteoarthritis cause a significant burden to both individuals and societies [1, 2]. LBP is estimated to be the leading cause of disability worldwide, and it is highly prevalent in working-age populations [2]. At the same time, the prevalence of obesity is increasing [1]. Overweight and obesity are associated with an increased risk of LBP and a need for treatment, especially in chronic cases [3].

Obesity can be proxied by various anthropometric measurements such as body mass index (BMI), waist circumference (WC), body fat percentage (BF%), waist-to-height ratio (WHtR) and waist-to-hip ratio (WHR) [4, 5]. Different indicators measure obesity in different ways, and classify individuals as obese on the basis of varying criteria.

In disc degeneration, the disc loses its elasticity and height [6]. Some previous studies [7–12] have suggested a significant association between lumbar disc degeneration (LDD) and LBP, although not all studies [13, 14] have obtained the same findings. This discrepancy highlights the need to clarify whether any underlying factors might modify the association between LDD and LBP. In our previous study, we found that the association between LDD and LBP-related disability depended on mental distress and insomnia status [15].

Previous studies have suggested that obesity is independently associated with both LDD and LBP [3, 16–19], although the majority of these studies have explored these associations using only BMI as an indicator. It is noteworthy that studies of the role of obesity as a potential modifier of the association between LDD and LBP are lacking.

## Methods

### Aim

This population-based study employed various methods to measure obesity, to comprehensively investigate whether the association between LDD and LBP-related disability would differ among individuals with and without obesity, i.e., whether obesity modifies the association. For this purpose, it used a large sample of Northern Finns reporting LBP. Our hypothesis was that obesity might modify the association between LDD and LBP-related disability. Our reason for studying this was that obesity has previously shown to be associated with both LDD and LBP/pain perception and we wanted to explore several obesity parameters that may modify the connection between LDD and LBP related disability [16].

### Study design

#### Cross-sectional study

##### Study sample

The Northern Finland Birth Cohort 1966 (NFBC1966) is a prospective longitudinal population-based cohort (*n* = 12058 live births). Pregnant women who lived in one of the two northernmost provinces of Finland (Oulu and Lapland) and whose expected date of delivery was in 1966 were invited to participate. The cohort participants consist of the live-born children of these women, and they have been followed since 1966, through regular postal questionnaires and clinical examinations, which have included objective measurements of weight and height [20, 21].

**Fig. 1:**
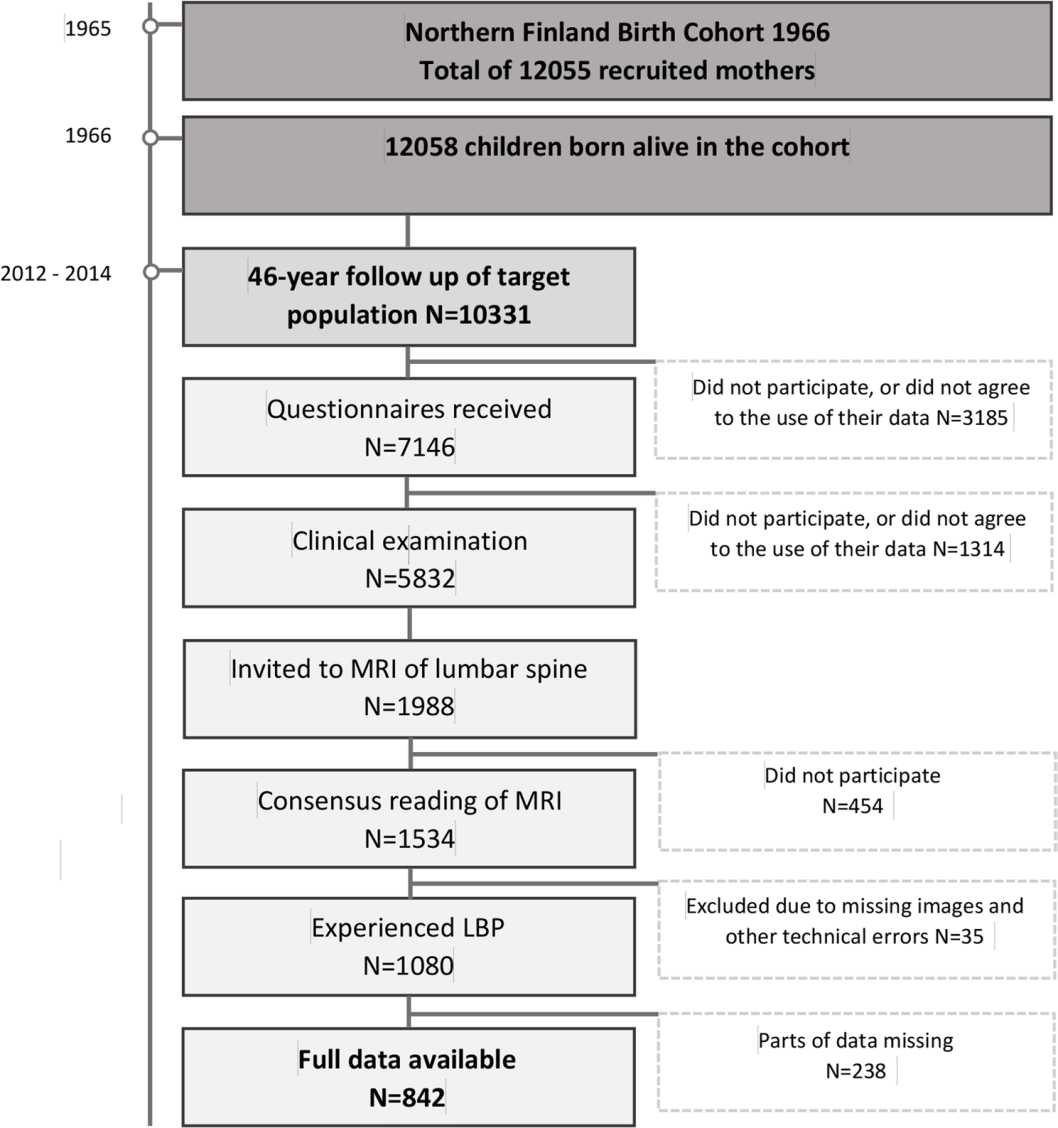
Flowchart of Northern Finland Birth Cohort 1966 study [20]. At each point, the target population consisted of cohort members who were alive and had a known address. Individuals who attended the clinical examinations and were living within 100 km of the city of Oulu (*n* = 1 988) were invited to undergo MRI. N, number; MRI, magnetic resonance imaging; LBP, Low back pain

The data used in this study were gathered using postal questionnaires and clinical examinations conducted in the most recent follow up, when the cohort members were aged 47, in 2012–2014. A subpopulation living in the area of Oulu, Finland underwent a more detailed clinical examination. A total of 1080 individuals who underwent lumbar MRI had experienced LBP during the previous year and the study sample was based on 842 of these individuals whose full data were available (Fig. 1).

### Assessment of lumbar MRI and evaluation of LDD

A total of 1540 NFBC1966 participants underwent an MRI of the lumbar spine in 2012–2014 (Fig. 1). The lumbar MRI used 1.5-T equipment (Signa HDxt, General Electric, Milwaukee, WI) and T2-weighted fast-recovery fast spin-echo (frFSE) images in the sagittal and transverse planes, and T1-weighed fluid-attenuated inversion recovery sequence images in the sagittal plane. A detailed description of the MRI protocol is reported in a previous publication [11]. The scans were evaluated using NeaView Radiology software (Neagen Oy, Oulu, Finland), version 2.31. Individuals who attended the clinical examinations and were living within 100 km of the city of Oulu (*n* = 1 988) were invited to undergo MRI.

A Pfirrmann-based LDD consensus reading was carried out, as has been previously described [11]. In the first stage, the MRI scans were independently assessed by experienced musculoskeletal radiologists (JN and RB) and an experienced physiatrist with an extensive history in spinal imaging (JK). In the second stage, the first author (TM) pursued consensus. The inter-rater reliability ranged from fair to good (κ = 0.39 to 0.79) and the evaluators were blinded to all other data and parameters used in the study [11].

The overall burden of LDD was quantified, using the final LDD consensus, by means of a Pfirrmann-based sum score variable by categorizing Grades I and II as 0, and Grades III, IV and V as 1, 2 and 3, respectively. The LDD sum score for five lumbar discs can theoretically range from 0 to 15, with higher values indicating higher LDD burden [11, 22].

### Assessment of low back pain

Data on LBP were collected using a questionnaire administered at the same time as the MRIs were performed [11]. The anatomical area of the LBP was illustrated by a drawing. LBP-related disability was defined on the basis of responses to the following questions: 1) ‘Have you had any aches or pains in your lower back within the last 12 months? (no / yes)’. If the response was ‘yes’, participants were asked about the severity of pain-related disability at work, during leisure time and during sleep (altogether), rating it on a numerical rating scale (NRS) from 0 (no pain) to 10 (extremely bothersome pain/prevents activity/total disability).

### Assessment of obesity

Obesity was assessed using the following five objectively measured anthropometric indicators: BMI, WC, BF%, WHtR, and WHR. As part of the clinical examination of the cohort, weight (kg), height (cm), WC (cm) (midway between the lowest rib margin and the iliac crest) and hip circumference (cm) (at the widest trochanters) were measured by a trained nurse. BMI, WHR, and WHtR were calculated as follows: BMI was calculated by dividing weight by height squared, WHR was calculated by dividing WC by hip circumference, and WHtR was calculated by dividing WC by body height. BF% was based on bioimpedance analysis (InBody 720, Biospace Co., Seoul, Korea) [23]. The cut-off values for the obesity indicators were determined on the basis of previous research and set at levels indicating a high or very high risk of obesity-related comorbidities. Each indicator was dichotomized as non-obesity and obesity according to these cut-offs. All body composition measurements were taken after a 12-hour fasting period.

The cut-off value for BMI follows the definition of the World Health Organization (WHO) for obesity: 30 kg/m^2^ [18]. The cut-off values for abdominal obesity measured by WC follow international standards: 102 cm for men and 88 cm for women [18, 24–27]. WHO’s WHR cut-off values are 0.95–1.0 for Caucasian men and 0.85 for Caucasian women [5, 27, 28]. As the vast majority of men would have been classified as having significant abdominal obesity by using the cut-off of 0.95, the cut-off for men was set at 1.0. For women, we used the recommended value of 0.85. The cut-off for WHtR was set at 0.6 for both genders [26, 29]. As BF% has no universal cut-off, we used 25% for men and 35% for women, based on the previous literature [30–32].

### Assessment of confounders

Previous studies have identified sex, smoking, education, leisure-time physical activity, occupational physical exposure, Modic changes and disc herniations observed in lumbar MRIs as potential confounders in the association between LDD and LBP [3, 17, 33–43]. Information on these variables was collected when the study participants were 47 years old.

Smoking was elicited by two questions in the questionnaire: (1) ‘Have you ever smoked cigarettes (yes/no)?’ and (2) ‘Do you currently smoke (yes/no)?’ Based on the responses, participants were classified into three groups: (1) never-smokers, (2) former smokers and (3) current smokers [44].

Education level was categorized according to the number of school years completed: <9 school years, 9–12 school years, > 12 school years, based on the Finnish education system and consistent with previous research [44].

To define physical activity during leisure time, the participants were asked how often they took part in physical activity that caused at least some sweating and breathlessness (corresponding to moderate-to-vigorous intensity). The response alternatives were (1) daily, (2) 4–6 times a week, (3) 2–3 times a week, (4) once a week, (5) 2–3 times a month, and (6) once a month or less often. The participants were divided into three categories: ‘active’ (active at least four times a week), ‘moderately active’ (active 1–3 times a week), and ‘inactive’ (active less than once a week) [36].

Occupational physical exposure was defined in the same way as that described in previous research [38]. On the basis of their responses, the participants were classified into two categories according to their occupational physical activity: ‘Low’ (high-intensity tasks, such as hard physical labour, constant moving, and lifting heavy loads, performed rarely or occasionally) and ‘High’ (at least one high-intensity task performed frequently).

The protocols for evaluating the presence of Modic changes and lumbar disc herniations have been described previously [11, 44]. The presence of disc herniations was assessed and dichotomized as ‘no disc displacement or bulge’, or ‘ protrusion, extrusion or sequester’ [44]. Modic changes were evaluated and dichotomized as ‘present’ or ‘absent’ [11, 44].

### Statistical analyses

Statistical analyses were performed using SPSS Statistics, version 27, 64-bit edition (IBM, Armonk, NY, USA). The threshold of statistical significance was set at *P* = 0.05. Descriptive statistics were used to present the distributions of LDD, LBP-related disability, obesity indicators and confounders in the final study sample; frequencies (n) and percentages (%) were used for the categorical variables; and means with standard deviations (SD) or medians with interquartile ranges (IQR) were used for the continuous variables. Distributions of LDD and LBP-related disability were also presented stratified by obesity indicators.

The association between the LDD sum score (continuous exposure) and LBP-related disability (continuous outcome) was modelled using general linear regression, with the beta coefficient (ß), 95% confidence interval (CI) and P value obtained from the data output. Here, β is interpreted as the change in LBP-related disability score per 1-unit increase in LDD sum score. Both unadjusted and fully adjusted models were constructed, and all analyses were conducted on a complete-case basis. To study the role of obesity in the associations between LDD and LBP-related disability, all the regression models were performed separately for individuals with and without obesity according to the five anthropometric indicators.

### Ethical approval

The study followed the principles of the Declaration of Helsinki and was approved by the Northern Ostrobothnia Hospital District’s Ethical Committee 94/2011 (12.12.2011). The NFBC1966 members took part voluntarily and signed their informed consent. All information on personal identity was encrypted and pseudonymized before being granted to the researchers.

## Results

### Characteristics of the study population

The study sample used in the main analyses consisted of 842 individuals with LBP-related disability whose full data was available (Table 1). Just over half of the sample were women (54.8%) and had never smoked (53.3%). Almost three quarters had 9–12 years of education (73.3%). Just over half of the sample engaged in leisure-time physical activity 1–3 times per week (56.8%) and had low occupational physical exposure (57.8%). The average LBP-related disability was 4.7 (SD 2.5) and the median LDD score was 4 (IQR 3–6). A breakdown of LDD sum scores and LBP-related disability scores in obesity groups is presented in Supplementary Table 1. The obesity indicators exhibited some degree of heterogeneity, classifying 15.6%, 19.5%, 33.1%, 36.1% and 40.7% of the sample as obese on the basis of their BMI, WC, BF %, WHtR and WHR, respectively. 

**Table 1:** Characteristics of final study sample with low back pain (*n* = 842)

Variable	% (*n*) unless otherwise indicated	
Sex		
Men	45.2 (381)	
Women	54.8 (461)	
Smoking		
Non-smoker	53.3 (449)	
Former	31.2 (263)	
Current	15.4 (130)	
Education years		
< 9	2.9 (24)	
9–12	73.3 (617)	
> 12	23.9 (201)	
Leisure-time physical activity (times/week)		
< 1	27.1 (228)	
1–3	56.8 (478)	
≥ 4	16.2 (136)	
Occupational physical exposure		
Low	57.8 (487)	
High	42.2 (355)	
LBP-related disability, mean (SD)	4.7 (2.5)*	
LDD sum score, median (IQR)	4 (3–6)**	
Modic changes		
Absent	27.4 (231)	
Present	72.6 (611)	
Disc herniations		
No disc displacement or bulge	79.6 (670)	
Protrusion, extrusion or sequester	20.4 (172)	
Body mass index		
< 30 kg/m^2^	80.5 (678)	
≥ 30 kg/m^2^	19.5 (164)	
Waist circumference		
M < 102 cm, W < 88 cm	66.9 (563)	
M ≥ 102 cm, W ≥ 88 cm	33.1 (279)	
Body fat percentage		
M < 25%, W < 35%		63.9 (538)
M ≥ 25%, W ≥ 35%		36.1 (304)
Waist-to-height ratio		
< 0.6	84.4 (711)	
≥ 0.6	15.6 (131)	
Waist-to-hip ratio		
M < 1.0, W < 0.85	59.3 (499)	
M ≥ 1.0, W ≥ 0.85	40.7 (343)	

*LBP* Low back pain, *LDD* Lumbar disc degeneration, *M* Men, *W* Women, *kg/m*^*2*^ Kilogram per meter squared, *cm* Centimetre, *%* Percent, *SD* Standard deviation, *IQR* Interquartile range, *n* Number

*Presented as mean (SD)

**Presented as median (IQR)

### Associations between LDD and LBP-related disability among participants with and without obesity 

A statistically significant positive association between LDD and LBP-related disability was identified among participants without obesity, measured using any of the five indicators (Table 2). The strongest association was observed when obesity was measured by BF% (unadjusted ß 0.205, 95% CI 0.122–0.287, p<0.001; adjusted ß 0.187, 95% CI 0.091–0.283, p<0.001). In contrast to these results, no significant associations between LDD and LBP-related disability were observed among the participants with obesity. These findings were consistent across both the unadjusted and adjusted models.

**Table 2:** Associations between lumbar disc degeneration sum score and low back pain-related disability, stratified by body mass index, waist circumference, body fat percentage, waist-to-height ratio and waist-to-hip ratio

Variable	Unadjusted ß (95% CI) (n=842)	Adjusted^1^ ß (95% CI) (n=842)
1. Body mass index		
<30 kg/m^2^	0.153 (0.075–0.231), p<0.001	0.119 (0.030–0.208), p=0.009
≥30 kg/m^2^	0.108 (-0.056–0.272), p=0.196	0.170 (-0.018–0.359), p=0.077
2. Waist circumference		
M <102cm, W <88cm	0.171 (0.086–0.255), p<0.001	0.134 (0.038–0.231), p=0.007
M ≥102cm, W ≥88cm	0.086 (-0.040–0.213), p=0.180	0.090 (-0.053–0.232), p=0.216
3. Body fat percentage		
M <25%, W <35%	0.205 (0.122–0.287), p<0.001	0.187 (0.091–0.283), p<0.001
M ≥25%, W ≥35%	0.038 (-0.090–0.166), p=0.559	-0.023 (-0.164–0.118), p=0.748
4. Waist-to-height ratio		
<0.6	0.179 (0.103–0.254), p<0.001	0.149 (0.064–0.235), p<0.001
≥0.6	-0.048 (-0.244–0.148), p=0.629	-0.014 (-0.251–0.223), p=0.908
5. Waist-to-hip ratio		
M <1.0, W <0.85	0.182 (0.094–0.270), p<0.001	0.171 (0.067–0.275), p=0.001
M ≥1.0, W ≥0.85	0.091 (-0.025–0.207), p=0.122	0.067 (-0.060–0.193), p=0.302

Statistically significant values are bolded

*B* Beta coefficients, *CI* Confidence interval, *n* Number, *M* Men, *W* Women, *cm* Centimetre, *kg/m*^*2*^ kilogram per meter squared, *%* Percent

^1^Adjusted for sex, smoking, education, leisure-time physical activity, occupational physical exposure, Modic changes, and disc herniations

## Discussion 

The aim of this population-based study was to explore whether the association between LDD and LBP-related disability would differ among individuals with and without obesity, i.e., whether obesity modifies this association. We found that obesity significantly modified the association between LDD and LBP-related disability: the association between increasing LDD and increasing LBP-related disability was statistically significant among individuals without obesity, but not among those with obesity. This finding was consistent across all the obesity indicators (BMI, WC, BF %, WHtR and WHR), and in both the unadjusted and adjusted models, despite the inclusion of confounders.

In this study, between 15.6% and 40.7% of the participants were classified as having obesity, depending on the anthropometric proxy, indicating heterogeneity across the measures. The definition of overweight or obesity depends on the amount and distribution of adipose tissue, which is why we used several indicators. The cut-off values used in this study were based on either standardized values or those commonly used in the existing literature. WC and BF% classified a larger proportion of individuals as having obesity, whereas WHtR and BMI classified a smaller a proportion of individuals as having obesity. BMI is the internationally accepted standard for estimating obesity and is the most widely used tool for measuring it [45]. However, it also has limitations. For example, it cannot separate fat mass from muscle mass and does not account for the distribution of fat in the body [18]. Previous studies have suggested that individuals with increased WC, WHR and BF% are more likely to experience LBP, regardless of their BMI status [46]. According to WHO’s age-standardized BMI-based estimates, the prevalence of obesity among Finnish adults was 21.5% (18.8–24.4) in 2022 [47]. A Finnish study has shown the prevalence of obesity among Finns to be 30% for women and 27% for men when BMI and WC are considered as indicators [48], and WHO estimates that nearly one in four (23%) adults in the European region have obesity [49]. These results are broadly in line with ours. Nevertheless, all definitions of obesity showed a similar modifying effect on the association between LDD and LBP-related disability.

 LDD has been identified as a significant risk factor for LBP [2, 11]. The association between LDD and LBP may be influenced by various factors. Independent positive associations have been found between obesity and both LDD and LBP [16], providing a basis for our approach exploring the modifying effect of obesity in the association between LDD and LBP. We observed a positive association among individuals without obesity across all the obesity measures in our data. The greatest contrast in point estimates between the obese and non-obese strata were observed using BF%, as also noted in a previous study [46], followed by WHR. The models were adjusted for several potential confounders, thereby enhancing the internal validity of our results. However, the lack of statistically significant associations in obese groups does not negate the relevance of LDD to disability; rather it may suggest the presence of competing underlying mechanisms such as metabolic stress.

Only two previous studies, also published by the authors of this paper, have examined the factors that modify the relationship between LDD and LBP [11, 15]. First, we found that depressive symptoms eliminated the positive linear association between LDD burden and LBP-related disability [11]. In the second study, we found that insomnia has a potential role in the equation, as the positive association between LDD and LBP-related disability was lost among individuals with co-occurring insomnia and mental distress [15]. The WHO European Regional Obesity Report 2022 [49] highlights growing evidence of an association between obesity and depression among adults. Based on both previous studies and the present findings, these factors appear to play a significant modifying role in the LDD-LBP association, but the underlying factors remain unknown. 

Although this study is cross-sectional and cannot determine causality, its findings indicate that LDD burden, as evaluated from lumbar MRI, may not be among the most relevant contributory factors of LBP-related disability among individuals with obesity. This would imply that other potential factors for LBP-related disability should also be deciphered in addition to LDD. These factors may be lifestyle factors and other degenerative spine conditions [2], depression [15], or obesity itself [2]. Our study suggests that LBP treatments tailored according to obesity status may be needed, but naturally, this cannot be shown in the current study context. A comprehensive approach addressing obesity-related factors, including weight management, may be useful for obese patients to improve their LBP outcomes [50]. Further research using longitudinal data and prospective designs, as well as clinical studies, could deepen our understanding of the relationships between LDD, obesity and LBP-related disability. 

### Strengths and weaknesses of the study

This study had several notable strengths. Firstly, we utilized a general population sample of Northern Finns who reported LBP, ensuring a relatively broad and representative cohort [21, 51]. Secondly, obesity was defined by means of various anthropometric proxies that were objectively measured in a clinical examination. Thirdly, LBP-related disability was selected as the primary pain dimension due to its comprehensive nature, capturing pain-related disability across various contexts, including work, leisure time and sleep [52]. The experienced experts reached LDD consensus. The evaluators were blinded to all the other data. Additionally, we adjusted for several potential confounders, which strengthens the internal validity of our results.

Our study also had limitations. Its cross-sectional design precludes the determination of causality or the potential directions of the associations, and the assessment of temporal relationships is not possible. There may be some limitations in the study samples representativeness due to attrition and limited geographic area from which the participants were recruited for the MRI. To enable the analysis, the concept of obesity was simplified using binary variables which showed some heterogeneity in classifying individuals as obese. Although the definitions of the cut-offs for obesity indicators other than BMI and WC were not universal, they were based on previous research and were set at levels indicating a high or very high risk of obesity-related comorbidities, which enhances their clinical relevance. Although a wide range of sociodemographic and lifestyle variables were considered in the study, certain factors—such as mental distress, insomnia, genetic predisposition and adipokine profiles—were not included. Future research is warranted to clarify their roles in the equation. Unfortunately, our dataset does not include information regarding the duration of the LBP episodes, and we acknowledge that the pathophysiology may differ between chronic and acute pain. However, LBP-related disability was selected as the primary pain dimension due to its comprehensive nature and high clinical significance among individuals with LBP. 

## Conclusions

This study investigated the role of obesity in the association between LDD and LBP-related disability among a large population-based sample of Northern Finns with LBP. Our findings suggest that LDD is associated with LBP-related disability among individuals without obesity but not individuals with obesity. Although this cross-sectional study cannot establish causality, the findings suggest that LDD burden, as evaluated by lumbar MRI, may not be among the most relevant factors of LBP-related disability among individuals with obesity. This would imply that other potential factors of LBP-related disability in addition to LDD should be investigated. While our results indicate that the observed association between LDD and disability might be weaker among individuals with obesity, the underlying mechanisms remain unclear. Further research with longitudinal data and prospective designs are warranted. In particular, future studies should aim to investigate the role of mental distress in the equation.

## Supplementary Information

Supplementary Material 1

## Data Availability

Availability of data and material: NFBC data are available from the University of Oulu, Infrastructure for Population Studies. Permission to use the data for research purposes can be applied for via an electronic material request portal. In our use of data, we followed the EU general data protection regulation (679/2016) and the Finnish Data Protection Act. The use of personal data is based on the cohort participant’s written informed consent in their latest follow-up study, which may limit its use. Please contact the NFBC project centre (NFBCprojectcenter@oulu.fi) or visit the cohort website (https://www.oulu.fi/nfbc) for more information.

## Abbreviations

B: Beta coefficient
BMI: Body mass index
CI: Confidence interval
BF%: Body fat percentage
IQR: Interquartile range
LBP: Low back pain
LDD: Lumbar disc degeneration
MRI: Magnetic resonance imaging
NFBC1966: Northern Finland Birth Cohort 1966
n: Number
NRS: Numerical rating scale
SD: Standard deviation
WC: Waist circumference
WHR: Waist-to-hip ratio
WHtR: Waist-to-height ratio
