# Supplementary material for: The role of co-occurring obesity in the association between lumbar disc degeneration and disability related to low back pain
Source: BMC Musculoskelet Disord. 2026 Jan 19;27:137. doi: 10.1186/s12891-026-09514-5 (PMC12895865; doi:10.1186/s12891-026-09514-5)
Supplement: Supplementary file 1 — Supplementary Material 1 [file 12891_2026_9514_MOESM1_ESM.docx]

Supplementary table 1. Lumbar disc degeneration (LDD) sum score and low back pain (LBP) -related disability distributions by obesity indicators.

| Variable | LDD sum score, median (IQR) | LBP-related disability, mean (SD) |
| --- | --- | --- |
| 1. Body mass index |  |  |
| <30 kg/m² | 4 (3–6) | 4.6 (2.4) |
| ≥30 kg/m² | 4 (3–6) | 5.0 (2.7) |
| 2. Waist circumference |  |  |
| <M 102 cm, W 88 cm | 4 (2–6) | 4.5 (2.4) |
| ≥M 102 cm, W 88 cm | 4 (3–6) | 5.0 (2.5) |
| 3. Body fat percentage |  |  |
| <M 25%, W 35% | 4 (3–6) | 4.5 (2.4) |
| ≥M 25%, W 35% | 4 (3–6) | 5.1 (2.6) |
| 4. Waist-to-height ratio |  |  |
| <0.6 | 4 (3–6) | 4.6 (2.5) |
| ≥0.6 | 5 (3–6) | 4.9 (2.6) |
| 5. Waist-to-hip ratio |  |  |
| <M 1.0, W <0.85 | 4 (3–6) | 4.6 (2.4) |
| ≥M 1.0, W ≥0.85 | 4 (3–6) | 4.9 (2.6) |

BMI, body mass index; LDD, lumbar disc degeneration; LBP, low back pain; IQR, interquartile range; SD, standard deviation; cm, centimetre; kg/m², kilogram per meter squared; M, men; W, women; %, percent.
